# Draft Genome Sequence of *Gordonia lacunae* BS2^T^

**DOI:** 10.1128/genomeA.00959-17

**Published:** 2017-10-05

**Authors:** Kim Durrell, Alaric Prins, Marilize Le Roes-Hill

**Affiliations:** a Biocatalysis and Technical Biology Research Group, Institute of Biomedical and Microbial Biotechnology, Cape Peninsula University of Technology, Bellville, South Africa; b Department of Microbiology, Faculty of Science, Stellenbosch University, Stellenbosch, South Africa; c Department of Biotechnology, Faculty of Natural Sciences, Institute of Microbial Biotechnology and Metagenomics, University of the Western Cape, Bellville, South Africa

## Abstract

We report here the draft genome sequence of the soil bacterium *Gordonia lacunae* BS2^T^ (= DSM 45085^T^ = JCM 14873^T^ = NRRL B-24551^T^), isolated from an estuary in Plettenberg Bay, South Africa. Analysis of the draft genome revealed that more than 40% of the secondary metabolite biosynthetic genes encode new compounds.

## GENOME ANNOUNCEMENT

Actinobacteria are an excellent source of novel biologically active secondary metabolites (1). This group of bacteria account for the production of over two-thirds of known secondary metabolites (2). A new species—*Gordonia lacunae*, with strain BS2 as the type strain—was described by Le Roes et al. in 2008 (3). The genome was sequenced using the Illumina MiSeq platform. A sequencing library was constructed with 1 ng of input DNA using the Nextera XT (Illumina) kit according to the manufacturer’s instructions, with the exception of the bead-based normalization step, which was omitted. Library quantification was performed using the Qubit HS assay (Invitrogen), diluted with Tris-HCl (pH 7.8), and pooled at 8 pM. The library was sequenced on an Illumina MiSeq sequencer using an Illumina MiSeq 600-cycle (2 × 300-bp) sequencing cartridge (V3). A 10% PhiX spike was included in the run to account for the high G+C content of actinobacterial DNA. The genome was assembled using the A5-miseq pipeline (4). Functional annotation of the predicted protein sequences was performed with the Rapid Annotations using Subsystems Technology (RAST) (5) and NCBI (6) servers. Secondary metabolite biosynthetic gene clusters (smBGCs) were predicted using antiSMASH (7). The draft genome sequence of *G. lacunae* BS2^T^ is 5,756,417 bp in length, with an average G+C content of 68.08%. The assembled genome has a coverage of 100× and an *N*_50_ size of 152.68 kb, consisting of 90 contigs with 5,102 coding sequences. Sixty-one RNA genes are found in the BS2^T^ genome, comprising 12 rRNAs, 46 tRNAs and 3 other RNAs. Strain BS2^T^ contains genes involved in processing and posttranslational modifications (apolipoprotein *N*-acyltransferase, lipoprotein signal peptidase, and prolipoprotein diacylglyceryl transferase) of bacterial lipoprotein precursors. The antiSMASH bioinformatics tool predicted 18 smBGCs, of which, there were 8 nonribosomal peptide synthetases (NRPSs), 1 NRPS-siderophore hybrid, 1 type I polyketide synthase, 1 bacteriocin, 2 terpene clusters, 1 aryl polyene cluster, 1 ectoine cluster, and 3 gene clusters labeled as “other.” Eight of the 18 smBGCs showed no homology to the biosynthetic pathways of known compounds curated in the antiSMASH database, one of which is the aryl polyene cluster. Bacterial pigments, such as the orange pigments produced by *Gordonia* spp., are a result of the expression of aryl polyene biosynthetic gene clusters. These pigments function as carotenoids that protect the bacterium from reactive oxygen species, thereby reducing potential oxidative stress-related cell damage (8). These results highlight the genetic potential of strain BS2^T^ for natural product discovery.

### Accession number(s).

This whole-genome shotgun project has been deposited in DDBJ/ENA/GenBank under the accession number NGFO00000000. The version described in this paper is the first version, NGFO01000000.
